# Becoming an Older Volunteer: A Grounded Theory Study

**DOI:** 10.1155/2011/361250

**Published:** 2010-12-27

**Authors:** Janet Witucki Brown, Shu-li Chen, Linda Mefford, Allie Brown, Bonnie Callen, Polly McArthur

**Affiliations:** ^1^Knoxville College of Nursing, The University of Tennessee, 1200 Volunteer Blvd, Knoxville, TN 37996-4180, USA; ^2^Caylor School of Nursing, Lincoln Memorial University, 6965 Cumberland Gap Parkway, Harrogate, TN 37752, USA

## Abstract

This Grounded Theory study describes the process by which older persons “become” volunteers. Forty interviews of older persons who volunteered for Habitat for Humanity were subjected to secondary content analysis to uncover the process of “becoming” a volunteer. “Helping out” (core category) for older volunteers occurs within the context of “continuity”, “commitment” and “connection” which provide motivation for volunteering. When a need arises, older volunteers “help out” physically and financially as health and resources permit. Benefits described as “blessings” of volunteering become motivators for future volunteering. Findings suggest that older volunteering is a developmental process and learned behavior which should be fostered in older persons by personally inviting them to volunteer. Intergenerational volunteering projects will allow older persons to pass on knowledge and skills and provide positive role modeling for younger volunteers.

## 1. Introduction

As the numbers of older adults in developed countries increase, it becomes increasingly necessary to view aging differently than we have in the past. According to U.S. statistics, 12.8% of the population was over the age of 65 in 2009. Further, individuals who reached the age of 65 could expect to live an additional 19.8 years if female, and an additional 17.1 years, if male [1]. Along with increased life expectancy is increased productive years. Policy focus worldwide is shifting from attention on dependency, frailty, and poor health commonly thought of as associated with aging toward a focus on healthy and productive aging and quality of life [2]. 

One indication of productive aging is volunteering. Older adult volunteering has become a topic for research and discussion in many countries including Spain, Hong Kong, Korea, and Australia [3–6]. Recent U.S. statistics reveal that 28.3% of older adults 55 to 64 years of age and 23.9% of those aged 65 and older volunteered in some capacity in the year 2008 [7]. While these numbers are encouraging, this also means that more than 70% of older American adults did not volunteer.

There have been numerous studies exploring volunteering in older adults in the last ten years. These studies have shown many benefits of volunteering to older adults, including: better physical and psychological health, increased well-being, increased life satisfaction, lower mortality risk, lower functional dependence, and lower levels of depression [8–12]. However, while benefits of volunteering for older adults have been well researched, it is not yet known how these older adults “become” volunteers. Understanding of why and how older adults volunteer is essential if service agencies hope to attract and increase their numbers of older volunteers. 

The purpose of this grounded theory study is to describe the process by which older adults become volunteers. “Become” as used in this study is a term describing how these older adults incorporate volunteering into who they are and not just what they do. It is our hope that understanding of how older adults become volunteers may lead to interventions to engage more numbers of older adults in volunteering.

### 1.1. Background

In an attempt to discover why and how older adults volunteer, many studies have explored common motivators regarding older adult volunteering. Altruism has been found to be one of the most significant motivating factors for older adults to volunteer [8–10, 13–15]. Other key factors include increased age [16], the need to increase self-esteem by feeling useful and productive [14], the need to fulfill a moral obligation to society [14], and the utilization of talent and expertise through volunteering and interacting with others [15, 17, 18]. Still other studies indicate more egoistic reasons for volunteering such as companionship [19], peer support and a sense of purpose and personal growth [20], continued productivity, acquisition of a new skill, a sense of personal satisfaction, and a response to a perceived sense of role loss [8, 10, 19]. Inclusive organizational cultures, flexible volunteer options, intergenerational volunteering opportunities, and opportunities for older unemployed and training programs have also been identified as incentives to volunteer [21].

Several studies have identified barriers that hinder older adult volunteering. Among barriers identified in the volunteering research literature are time commitment and financial constraints [8, 10, 15, 21]. Sometimes, older adults identified that they were not as involved as they would like to be due to involvement in other activities and responsibilities [18], or they had concerns about being overcommitted [15]. A study by Jirovec and Hyduck [22] revealed that low income and lack of education prevented some adults from taking part in the volunteer experience. Other authors, such as Mutchler et al. reported that some older adults identified difficulty finding opportunities that were appealing and flexible [23]. Further, the culture of organizations may also be a contributing factor to a lack of older volunteers. Some organization cultures caused concerns over age discrimination, and other organizations' preference for younger volunteers and nonwelcoming attitudes towards new volunteers were cited as barriers to volunteering in one study [21]. 

 Previous studies identifying motivating and hindering factors of volunteering have provided valuable information in understanding older adult volunteers. However, these studies focused on specific factors such as benefits, motivating factors and barriers to volunteering, not on the process of volunteering. This study proposed to address that gap by exploring the process of becoming an older volunteer. Understanding of the process of how an older adult becomes a volunteer will provide a more complete and dynamic viewpoint of older volunteering. 

### 1.2. Theoretical Perspective

In grounded theory research, the theory is derived from the words of the participants. The theory is not derived from another theoretical framework. However, no research is atheoretical. Researchers always possess a perspective or viewpoint of reality. As nurse researchers, we were guided by a nursing theoretical perspective that uniquely framed, but did not dictate the findings, in this study. The theoretical perspective for this study was Margaret Newman's theory of health as expanding consciousness [24]. Within this theoretical perspective, consciousness is defined as the informational capacity of a person to interact with the environment and can be seen in the quality and diversity of interactions between persons and their environment. Expanding consciousness is viewed as a process whereby a person becomes more of oneself, finds greater meaning in life, and reaches new connectedness with other people and the world in which they live [24]. 

There are choice points or factors in the lives of individuals that represent opportunities for personal growth and facilitate change in person-environment interactions. This growth and change is reflected in patterns of expanding consciousness such as increased relatedness with others or a higher being. Patterns will vary according to the unique configuration of each person-environment situation. However, the theory of health as expanding consciousness posits that there will be similarity of patterns among people who share similar life experiences. This study sought to identify those common patterns. Within this framework, increased interactive patterns of interpersonal relationships such as those formed during volunteer work can be viewed as a manifestation of expanding consciousness. The theory of health as expanding consciousness provided a perspective for interpreting the accounts of the participants and identifying patterns of interaction, thus providing a framework interpreting the development of the emerging theory. Volunteering, in this study, was viewed as a pattern of expanding interactions with others and therefore a manifestation of expanding consciousness.

## 2. Methods

We used a grounded theory secondary data analysis method for this study. After removing any identifiers from original transcripts from an earlier study of a convenience sample of 40 older individuals who volunteered for a Habitat for Humanity “blitz build” [25], the data were subjected to secondary analysis to uncover a substantive theory of becoming an older volunteer. The original mixed-methods study [25] used qualitative interviews to learn about health, functioning, motivation, culture, and volunteering patterns of older volunteers. All participants in the earlier study were asked open-ended questions regarding their volunteering activities past and present, earliest memories of volunteering, perceived benefits and barriers to volunteering, how they chose volunteering activities, and why they volunteered during 60-to-90-minute face-to-face interviews. Original interviews were conducted during the early fall of 2006, and participants gave permission for the data to be used in other studies when they consented to the initial study. University Institutional Review Board (IRB) approval was obtained for the earlier study and this secondary analysis. 

To put the original study in context, a Blitz Build is an intense, physically challenging activity during which a group of volunteers come together through Habitat for Humanity to build a complete home within a week. Habitat for Humanity International is an organization that uses volunteers of all ages to help build affordable houses for those in need. They operate all over the world in 90 countries, all 50 U.S. states, the District of Columbia, Guam, and Puerto Rico [26]. 

This secondary data analysis study used grounded theory methodology to inductively develop a theory that was grounded in actual experiences and faithful to everyday reality as conveyed by the actual participants' accounts of those events. Grounded theory was chosen because it is especially useful for uncovering process. This method was developed by sociologists for the purpose of “discovery of theory from data systematically obtained from social research” [27, page 2]. It is particularly useful for the understanding of fundamental social-psychological patterns [28] such as volunteering. Uncovering the process of “becoming” an older volunteer has the potential to lead to identification of methods to recruit and affect the process in other older adults thereby possibly increasing numbers of older volunteers. 

Data analysis was carried out in accordance with the techniques recommended by grounded theory researchers, Strauss and Corbin [29]. All qualitative data analysis was conducted using a team approach, with research team members consisting of faculty and graduate students from the University College of Nursing, coming together to read, code, analyze, and develop the emerging theory. The analysis process initially involved line-by-line reading of transcripts and identification of significant words or phrases (open coding) in order to break the data into pieces for the purposes of close analysis. Focused coding that is designed to identify and clarify larger more prominent concepts followed this process. Axial coding was utilized to connect the illuminated concepts and construct theory. A core category was identified, and all other categories were related to it by means of a paradigm model that identified context, antecedents, actions/interactions, intervening conditions, and consequences in order to fully capture the process. Finally, a story line describing the process was written, and the theory was depicted by a diagram. Throughout analysis, emerging findings were constantly compared to participant's words to assure that the findings were grounded in the data. Truthfulness of the theory was obtained through adherence to methodology, constant comparison of findings to participants' words, and member checking with verification of the finalized theory with several participants.

## 3. Results and Discussion

### 3.1. Sample Description

The sample for the original study [25] consisted of 24 men and 16 women (*N* = 40) from eleven southeastern states in the U.S. Participants were mostly Caucasian (92.5%). Three participants were Hispanic or other (7.5%). Mean age of participants was 68.64 years (range = 57–88; SD = 6.27). Also, the majority of participants were married (87.5%) and, in most cases, married couples came together to the Habitat Build. Most participants were semiretired (70%), but still working parttime. Eight participants (20%) were still employed fulltime. Many participants had a high-school or higher level of education (82.5%). 

### 3.2. A Grounded Theory of Older Adult Volunteering

**Figure 1:**
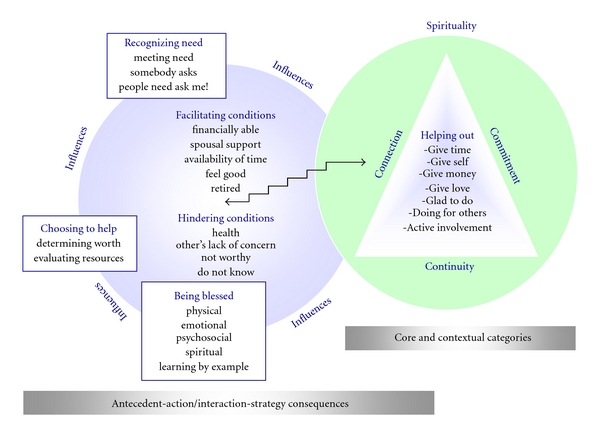
Theory of “becoming” an older volunteer.

Through analysis of the transcripts from the 40 participants of the original study, a theory of *Becoming an Older Volunteer* was discovered. The grounded theory is depicted in Figure 1. Readers may wish to refer to the diagram while reading the story line with exemplars that follows. Pseudonyms are used for all exemplars to protect anonymity of participants.

**Core Category**

When first asked about volunteering activities, the participants often responded that they did not volunteer much, however, they “helped out here and there” *Helping out* was the term they used for their volunteering activities, and this term became the core category of our theory. The older volunteers did not apply the term “volunteer” to themselves, rather they saw themselves as people who “help out.” It was apparent that *helping out *described common patterns of interaction by the participants with others. They also talked about years of *helping out* that began back in childhood. This helped us to conceptualize becoming a volunteer as a developmental process that evolves from helping out. Judy, a 65-year-old woman who was very active in her church, described how she helped out:

“I help at my church. I help in the dining room when there is a death in a family. I prepare a dish and I've gone and worked in the kitchen and helped serve the meals… I take older friends to their doctor's appointments. That's my routine now. You know, more just helping out, wherever.”

Another older woman, 70-year-old Susan, also described her church related activities:

“I'm treasurer of my Sunday School and on the finance committee… and my niece has a group of kids she calls “Bible Buddies” and I got started with them. I just thought it was a good idea for me to be there… So, I've been there to just help out with them, anything I can.”

**Contextual Categories**

There were three interacting and overlapping contextual categories named *continuity*, *connection,* and *commitment* that formed a contextual background for the core category. All three categories had a spiritual thread interwoven throughout. When taken together, this contextual background formed the motivational basis for the older volunteer to participate in *helping out.* Each of these contextual categories will now be discussed.

A desire to transmit values of caring to others that participants had learned earlier in their lives and their desire to pass on blessings they had received were named *continuity *in the theory. Many participants related early childhood memories of working together with their parents to help out needy neighbors or do church work. *Connection* described participants' sense of kinship with others and their love of people. These older adults felt a strong connection to others, generally, to all humankind, and specifically, through their churches and organization involvements. Belonging to a group that was committed to volunteering was a strong motivator for the participants to volunteer. *Commitment* described the participants' sense of personal responsibility to help others. For many, this sense of commitment had roots in their Christian beliefs. There was a sense of duty to help others expressed by almost all participants. Jeannie, an 80-year-old woman with a life-long connection to the Catholic Church described *helping out* in her life:

“I helped my mother… My mother… was the church cleaner in the Catholic church… so I think that's where I got my volunteering from.” *(continuity) *

Tom, a 68-year-old who grew up in a rural community, shared how this previous experience influenced his *helping out*:

“Most of the folks working with our bunch… grew up in the era where you helped your neighbor too.” *(continuity) *

Joe, 72 years old, and 40-year member of a fraternal organization that encouraged volunteering by members, described how belonging to the organization influenced his decisions in *helping out*:

“They are like a bunch of brothers, you know… and it's just a good feeling to be part of (civic) group.” *(connection) *

He also went on to discuss his own commitment and sense of personal responsibility to help others:

“I kindda feel a personal responsibility… I just feel led. That's what I need to do… I am my brother's keeper.” *(commitment) *

This sense of connection to others and a commitment arising from spiritual beliefs was echoed in these two statements by Mike, a 68-year-old retired life insurance salesman:

“I love people and I've never had the heart to say no, I guess, so I volunteer when I can.” *(connection)*… “God's got a whole lot for me to do and I feel like this is a part of it.” *(commitment) *

**Antecedent**

Before the process of *helping out* could begin, an antecedent event had to occur. The antecedent to the entire process was *recognizing a need*. This recognition of a need could occur in two ways: either an organization or individual asked for help or the older adults saw a need that they felt that they could meet individually or with others. Some participants were reluctant to help unless personally asked to do so, while others actively sought our opportunities to help others. Ron, a 70-year-old retired welder described how he recognized opportunities for *helping out*:

“Well, I, I've learned that I have to fill in where it needs to be filled in… I'm willing to do it.”

Ron's wife, 69-year-old Julie, described how she was willing to help out and anticipated being asked to help out every day:

“I've found out—what's the use of planning out your day? As soon as the first telephone rings, you know, the whole day is gone because there is somebody that needs me. I just learned not to—you just do every day what needs to be done if there is a need and you see it. I don't think you would think twice about doing for your neighbor, your friend. Whoever in need… I've never been any other way.”

Jerry, an 82-year-old man who was less outgoing, did not actively seek opportunities to help out. He described his reluctance in help out unless he was asked to do so:

“If somebody says “I need your help”, I'll help em. If they don't, why I don't butt in. That's it!”

**Action/Interaction Strategy**

Once a need was recognized, participants begin a deliberative process of *choosing to help*. The process of *choosing to help* consisted of several steps: First, the older adult determined the worthiness of the request or person needing help. Then, older volunteers evaluated their resources and abilities to give help. Finally, they determined the type of help they could give which could be financial, physical, or supportive help or sharing of knowledge, goods, or skills. Mary, a 65-year-old who assisted several neighbors who were much older than she was by running errands and taking them to appointments described her feelings about choosing to help out:

“But these are genuine people that need help. They know they can depend on me.”

Tom described his past personal experience of being poor and needing help himself at one time and how it influenced his decisions to *help out*:

“I love to help people that, you know, that I feel like they appreciate it and they need it. Because I came from being very poor… so now if I see somebody that I know that they need help, that's got several children and they've become divorced or widowed—I know they need help, you know.”

**Intervening Conditions**

Factors or conditions that could be either hindering or facilitating determined whether or not help was given, and also what kind of help was given. These intervening conditions included, but were not limited to: time to participate in a project or help out, financial resources, need for, and ability to travel, physical abilities or disabilities and general health status, skills and spousal support of the person volunteering. Several of the older adults, especially those 70-years old and older spoke of physical limitations which negatively impacted either the amount or type of volunteering they did. However, many participants considered volunteering to be a priority, and they continued to volunteer, but adapted what they contributed physically by adapting activities to their physical conditions. This area is addressed more completely in another publication [30]. Rebecca, a 70-year-old who frequently volunteered with her husband Harry who was 72 years old, related how having time to volunteer facilitated their ability to do so:

“I mean to us now we feel that we can do it now because we both don't have to say “I have to be home” or “I have my children”. They are all gone and they are all grown up so I feel I'm on my own now.” *(facilitating time) *

Sam, a widower and the oldest participant at 88, described how financial stability allowed him to be able to volunteer:

“As I say, I'm pretty frugal and I invested a little bit… So I have a fairly good little income and I'm glad to share it with something that I think will do some good.” *(facilitating finances) *

Jim, a 74-year-old who was anticipating a need for a hip replacement, described how this placed physical limitations on him and influenced his ability to help out:

“I can't get down, you know, low, because you know, it's hard for me to get back up because of my hip. Ain't actually my hip—it's the muscle in my leg. But uh, yeah, I wish I could do more, but I'm limited in, you know, that way.” *(hindering health condition) *

**Consequences**

The result (consequences) of *helping out* was described by participants as *being blessed*. The words *blessed or blessings *were used frequently by participants when they described benefits of volunteering. Participants related these as general blessings or as spiritual, social, emotional, or physical benefits gained as a result of the helping out experience. More importantly, these blessings reinforced their volunteering and strengthened the contextual categories of *continuity*, *connection, *and *commitment,* increasing the likelihood that the older volunteer would volunteer again.

Blessings also increased the likelihood that these older volunteers would actively seek future opportunities to volunteer and choose to help if asked. In fact, the more they helped out, the more they wanted to help out. Julie related blessings she received from volunteering:

“… you do something that they ask you to do and… you are accomplishing something and you feel good about yourself.”

Sam stated that he looked every day for an opportunity to help others. He related:

“It just feels so good to get to share some of the blessings that you had in your life… and I would definitely recommend this to anybody that wanted a blessing out of life. I feel blessed and it is good to share some of your blessings with somebody else.”

Ralph, a 69-year-old who was battling his own health problems (lung cancer), but still continued to volunteer whenever he was able, related the following:

“I guess, you know, it all just boils down to personal satisfaction. There is not a tremendous amount, I mean, well, there's no monetary really, I don't guess that I know of, but I just enjoy helping people and being with people and working with them.” (Ralph passed away a year later).

*Blessings,* in the form of physical and mental benefits of volunteering, were discussed by Carl, a very active 82-year-old.

“Well, it will probably make you get more exercise than you would ordinarily. So that helps you, physical exercise and mind and everything else.”

### 3.3. Discussion

As a result of analysis, it became very clear to us that the term “volunteer” described not only what these older volunteers did, but also who they were in a wholistic sense. These older adults incorporated volunteering as an integral part of their personal, emotional, and spiritual identities. The Habitat for Humanity Blitz Build was only one of many volunteering activities that the older participants engaged in. They related stories of volunteering in many different capacities both on a formal basis through organizations and on an informal level through work in their communities and with friends and family. Volunteering activities included work with church and civic organizations, assisting in voter registration and poll work, church mission work in the U.S. and other countries, disaster relief work, food ministry work, helping with disabled children, and many other instances. Further, these participants volunteered often, sometimes on a daily basis.

Our research shows that volunteering is a developmental process and a learned behavior. This finding implies that intergenerational volunteering projects should be encouraged, since volunteering is best learned by example. Intergenerational projects will allow older adults to pass on knowledge and skills and provide positive role modeling for younger volunteers. Additionally, younger volunteers can assist older adults with some aspects of volunteering projects that may require more physical strength or agility. Nurses, especially Parish Nurses, are in a unique position to assist in developing faith-based volunteering projects that include both older adults and youth members working together. Volunteering projects that involve several generations support Warburton et al.'s [21] findings that intergenerational opportunities are desired. 

Previous volunteering experience is not a prerequisite for an older adult to become a volunteer. A sense of *commitment, connection, or continuity* (context), however, is a strong motivator. Older adults, even without previous volunteering experience, can and should, personally be invited to volunteer. Older adults will more likely volunteer if they belong to a group or organization that volunteers. Our findings support previous studies that show that connection with groups is a motivating factor [3, 14, 19]. Nurses, through their work with churches and other civic groups, should encourage inclusion of older adults in organizations that have a variety of ongoing volunteer projects available. 

Also, positive volunteering experiences lead to the desire to volunteer more. Once an older adult has volunteered, additional positive volunteering experiences will lead to more volunteering (blessings). Projects where older adults can see the “fruits of their labors” and experience increased social contact will provide more “blessings” and lead to increased volunteering. Nurses should promote volunteering as an activity choice for older adults. The physical and mental benefits of such activities have been well documented in the literature and are supported in this study.

Our findings regarding intervening conditions support findings in other studies [8, 10, 15] that factors such as time, health, and finances can act as barriers to volunteering for older persons. However, while the time factor may have been a barrier to volunteering earlier in their lives while they were rearing children and involved in careers, many of our participants reported having increased time to volunteer because of retirement or semiretirement. Also, once a commitment was made to volunteer, the time was found since *helping out* became a priority. Health, unless it impacts functioning significantly does not seem to affect older adult volunteering either in this study or in the literature. Older adults will find a way to volunteer within their abilities, and nurses can assist older adults in finding volunteering opportunities that are congruent with the older person's physical abilities. Further, for volunteer activities where there may be a number of older adults participating, nurses can volunteer themselves to be the on-site nurses to provide first aide, check blood pressures, and assist with health related issues. 

Another barrier, that of limited finances, especially those related to transportation, may hinder lower-income older adults' ability to volunteer. Nurses can assist lower-income older adults with finding free or low-cost transportation through local Offices on Aging or other volunteer transportation services which would increase the ability of older adults to volunteer. Flexibility of scheduling while addressed in the literature [23] was not discussed by our participants as a problem or an incentive. However, volunteer opportunities that allow older adults to participant on a “drop-in” type of schedule would be more attractive and increase sustained participation by this age group. Habitat for Humanity is almost always involved in at least one building at any given time, and they encourage participants of all ages and at any time. 

When our findings are viewed within the Newman [24] perspective of health as expanding consciousness, the antecedent in the theory of *recognizing a need* represents a choice point for the participants to expand their interactions and connections with others. *Helping out *becomes a new pattern of relating for these participants in their older years because of more time when they retire and also financial ability, physical health, and spousal support. It becomes apparent that older adults who become volunteers have experienced expanding consciousness though increased sense of *connection *and interactions with others. These increased connections and the personal growth described by the participants were experienced as *blessings *in their descriptions of the benefits they derived from volunteering. In addition to increased *connection* with others, participants described how volunteering strengthened their spiritual *connections*. All participants felt that they were better people because of their volunteering activities. Thus, we conclude that volunteering leads to expanded consciousness as defined by Newman [24]. 

This new theory also supports Register Theory by Register and Herman [31]. The Register Theory posits that connectedness is paramount in older life and suggests that the tenets of connectedness produce quality of life in older adults: metaphysical connectedness, spiritual connectedness, biological connectedness, connectedness to others, and environmental connectedness. Our theory of becoming an older volunteer highlights the importance of *connection* to the volunteering of older adults both as a contextual motivating concept and as a consequence of volunteering which includes blessings or benefits of connectedness to others and spiritual connectedness. These patterns of connectedness contributed to satisfaction and quality of life in the older volunteers in this study. 

 There were some limitations to our study including small convenience sample size (*n* = 40), all participants being from the same geographic area, minimal ethnic diversity in the sample, and all participants belonging to the same organization. Additionally, all participants self-identified as Christians. These limitations influence transferability of the findings to similar groups of older volunteers. We hope to address these limitations through future studies with larger samples, in other geographic locations and with more culturally diverse groups. Additionally, other religious groups have humanitarian and volunteer projects and future studies should include older Jewish, Buddhist, and Muslim volunteers. 

## 4. Conclusions

This paper has implication for nurses, as change agents in their community roles to foster active recruitment and involvement of older volunteers who are a valuable, largely untapped resource. Older adults who are recruited will likely respond, and as they gain positive benefits from volunteering, they will continue to participate in volunteering activities. All communities can be stronger because of older volunteers. These older volunteers can provide valuable needed services that communities cannot afford to have if they have to pay for them. Nurses can act as spokespersons for older adults and assist with education of organizations and individuals regarding the contributions that older adults can make and foster a more positive attitude towards aging. 

McBride [32] challenges all communities to develop ways of engaging all older adults who have the interest to be engaged. She offers an “institutional capacity perspective” for development of civic roles for older adults that takes into account interests, abilities, and capacities. Approaches such as these could help to increase numbers of older volunteers. Nations cannot afford to undervalue the resource they have in potential older volunteers. Public officials and agencies will find it necessary to be creative in attracting and retaining older volunteers, especially those with lower incomes [33].

This study has provided new important information about the process of how older adults become volunteers. Understanding of this process and how it develops may lead to strategies that better engage older adults in volunteering and, more importantly, strategies that *keep *older adults volunteering, thereby leading to increased health for the older volunteers and many benefits for the communities in which they live.
